# Cognitive Assessment and Rehabilitation for Pediatric-Onset Multiple Sclerosis: A Scoping Review

**DOI:** 10.3390/children7100183

**Published:** 2020-10-15

**Authors:** Wei-Sheng Lin, Shan-Ju Lin, Ting-Rong Hsu

**Affiliations:** 1 Department of Pediatrics, Taipei Veterans General Hospital, Taipei 11217, Taiwan; romberg@gmail.com; 2 Department of Physical Medicine and Rehabilitation, National Taiwan University Hospital Yunlin Branch, Yunlin 640, Taiwan; y00964@ms1.ylh.gov.tw; 3 Institute of Clinical Medicine, National Yang-Ming University, Taipei 112, Taiwan; 4 Faculty of Medicine, National Yang-Ming University, Taipei 112, Taiwan

**Keywords:** cognition, cognitive rehabilitation, pediatric multiple sclerosis

## Abstract

Cognitive impairment is increasingly recognized as an important clinical issue in pediatric multiple sclerosis (MS). However, variations regarding its assessment and remediation are noted in clinical arena. This scoping review aims to collate available evidence concerning cognitive assessment tool and cognitive rehabilitation for pediatric MS. We performed a systematic search of electronic databases (MEDLINE, PubMed, CINAHL Plus, and Web of Science) from inception to February 2020. Reference lists of included articles and trial registers were also searched. We included original studies published in English that addressed cognitive assessment tools or cognitive rehabilitation for pediatric-onset MS. Fourteen studies fulfilled our inclusion criteria. Among them, 11 studies evaluated the psychometric aspects of various cognitive assessment tools in the context of pediatric MS, and different neuro-cognitive domains were emphasized across studies. There were only three pilot studies reporting cognitive rehabilitation for pediatric-onset MS, all of which used home-based computerized programs targeting working memory and attention, respectively. Overall, more systematic research on cognitive assessment tools and rehabilitation for pediatric MS is needed to inform evidence-based practice. Computer-assisted cognitive assessment and rehabilitation appear feasible and deserve further studies.

## 1. Introduction

Multiple sclerosis (MS) is a chronic central nervous system disorder characterized by inflammatory demyelination and neurodegeneration, and around 3–5% of patients have their disease onset prior to adulthood. Although physical disability is rarely seen in the first decade of disease course in pediatric-onset MS (POMS) [1], cognitive impairment is fairly common in this patient population. Findings across studies showed that around one-third of pediatric MS patients suffer from some degree of cognitive impairment, and it could be detected as early as nearing disease onset in a subset of patients [2,3,4,5]. Multiple cognitive domains have been reported to be affected in pediatric MS, including information processing speed, attention, working memory (WM), verbal and visuospatial memory, executive function, visuo-motor integration, and aspects of language function [4,5,6,7,8,9,10]. While reports of cognitive profiles of POMS have been accumulating, direct comparisons between these studies are often hampered by differences in patient characteristics and assessment tools. Indeed, it was noted that the results of cognitive evaluation might vary with the instruments used. For instance, Wuerfel et al. used several tests to tap WM and found that only more cognitively demanding tasks revealed group-level difference in WM performance between POMS and controls [6]. This exemplifies the importance to clarify the applicability and performance of various cognitive assessment tools in this patient population.

Despite growing awareness of cognitive issues in pediatric MS in recent years, there has been limited information to date concerning ways of cognitive remediation for these patients. Rehabilitative strategies for POMS are often extrapolated from those for adult MS in the real-world situations, but this approach needs validation. To obtain a panorama of this emerging field, we here seek to collate existing evidence on cognitive assessment tools and cognitive rehabilitation for POMS, which may serve as the basis for future directions of research and clinical practice.

## 2. Materials and Methods

We followed the methodological framework developed by Arksey and O’Malley [11], and this scoping review adheres to the Preferred Reporting Items for Systematic Reviews and Meta-analyses Extension for Scoping Reviews (PRISMA-ScR, Supplementary File S1) [12]. This study was retrospectively registered at Open Science Framework database (https://osf.io/uyd2q/) on 12 April 2020, and the review protocol is presented in Supplementary File S2.

### 2.1. Literature Search, Screening, and Selection

We conducted systematic literature searches in the following databases: PubMed, MEDLINE, CINAHL Plus, and Web of Science. Date of publication was not restricted (from inception to February 2020). The terms used in the searches were: “multiple sclerosis” AND (“pediatric” or “paediatric”) AND (“cognitive” or “cognition”). The most recent search was executed on 21 March 2020. The titles and abstracts of retrieved articles were then screened for relevance to cognitive evaluation and/or cognitive rehabilitation. The inclusion criteria were: (a) peer-reviewed original studies published in English; (b) studies that specifically addressed either cognitive assessment tools or cognitive rehabilitation for POMS. The exclusion criteria were: (a) articles that were either not peer-reviewed (e.g., book chapter) or not reporting original studies (e.g., review paper); (b) studies that aimed to characterize the cognitive profile of POMS, rather than to examine the performance and applicability of cognitive assessment tools or the effects of cognitive rehabilitation in this patient population. Every effort was made to obtain the full text of all potentially relevant articles, which were examined to determine the eligibility. We also screened the reference lists of relevant articles. The above process was independently carried out by two of the authors (W.-S.L. and S.-J.L.), and discrepancies were resolved by discussion with the senior author (T.-R.H.) and consensus among the authors.

On the other hand, we also searched ClinicalTrials.gov, European Union Clinical Trials Register, and Open Science Framework database for trials or projects pertinent to the themes of our review.

### 2.2. Data Charting

The included articles were read by two of the authors (W.-S.L. and S.-J.L.), and relevant data were charted and tabulated. For studies evaluating cognitive assessment tools, we extracted the year of publication, the tests (and subtests, if applicable) of interest and their targeted cognitive domains, the characteristics of study participants (such as sample size of disease and control groups, demographic and disease-related features), and main findings (particularly in relation to psychometric performance). For studies evaluating cognitive rehabilitation, we extracted the mode of intervention (including its frequency and duration, requirement of supervision, and targeted cognitive domains), study design, the characteristics of study participants, effects of intervention (including effects on targeted and non-targeted domains, and sustainability of effect), and factors associated with outcomes.

For relevant clinical trials identified from trial registers, the principal investigator, the aim and the design of the trial, and other relevant information were collected.

## 3. Results

### 3.1. Original Studies Evaluating Cognitive Assessment Tools for Pediatric MS

**Figure 1:**
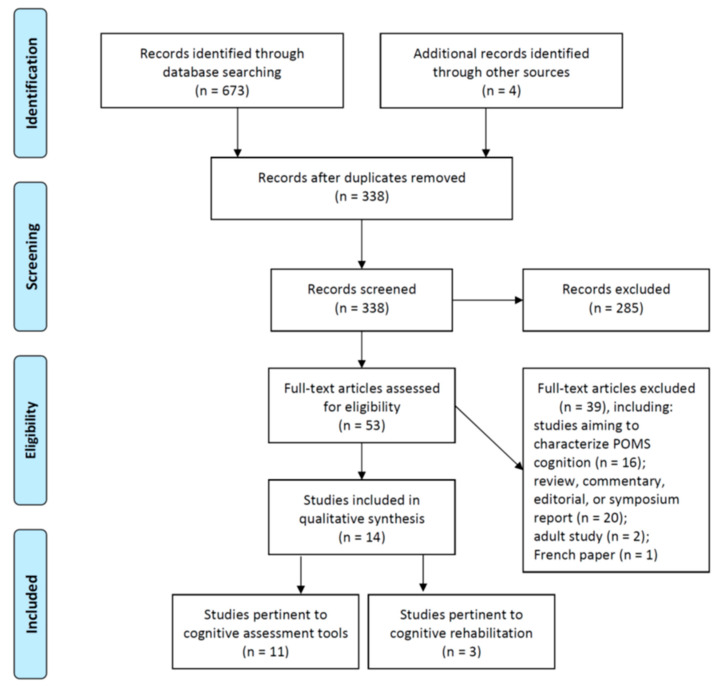
Diagram showing the workflow of literature search and selection process.

The workflow of this scoping review is shown in Figure 1. We identified eleven original papers evaluating the performance of cognitive assessment tools in the context of pediatric MS, with the earliest one published in 2009 [13]. A summary of these articles is provided in Table 1. These studies were largely cross-sectional in design. They were either single- or multi-centric, and all were carried out in North America and Europe. The focus of these studies differed from one another. Some studies tried to establish normative data using regression-based approach [14,15,16], in which age-squared variable could be incorporated to better model the nonlinear quality of cognitive development [15,16]. Others investigated the performance of various cognitive assessment tools through comparisons between patients and healthy controls [7,13,15,16,17,18,19,20,21]. Among these, two studies aimed to construct batteries by picking up three to four tests with better discriminating abilities, and evaluated the performance of these batteries as screening tools [13,21]. Participants’ satisfaction with the test was quantitatively reported in a study [17]. One study examined the interrelationships between tests tapping different cognitive domains [8]. A recurring finding yielded by these studies was the significant role of age and educational level in cognitive task performance in pediatric populations [14,17,21]. For instance, the symbol digit modalities test (SDMT) performance steadily improves with age in healthy children (8–17 years) [17]. On the other hand, older age predicted poorer SDMT performance in POMS after adjustment for disease severity (i.e., the expanded disability status scale, EDSS) [18]. Together these suggest divergent cognitive trajectories between normal children and pediatric patients with MS.

### 3.2. Original Studies Evaluating Cognitive Rehabilitation for POMS

We identified three original studies evaluating the effects of cognitive rehabilitation for POMS [22,23,24]. Their study design was summarized in Table 2. All three studies comprised interventions using home-based, computerized cognitive training. The targeted cognitive domains were working memory [22,23] and attention [24], respectively. The duration of a single training session was similar across these studies (45 min to 1 h), while the intensity varies from twice to five times per week. These studies were all pilot and exploratory in nature. The sample size was small (5–16 patients) and was not preplanned based on power analysis. No healthy control group was included in these studies. The study on attention retraining was a double-blind randomized clinical trial, using nonspecific training as the comparator arm. There was no comparator arm in the other two studies, hence the role of practice effect in neuropsychological evaluation cannot be clarified. The outcome measures included not only targeted cognitive function but also more extensive neuropsychological performance [23,24], and aspects of feasibility (adherence and tolerance to the training program) were evaluated as the main outcome in one study [23].

The results of these studies were summarized in Table 3. The study on attention retraining showed not only positive effects on attention and related cognitive domains, but also far transfer effect on visuospatial memory [24]. On the other hand, the other two studies, both focusing on working memory training, showed only modest effect on objective working memory measures, at group level. The far transfer effect was either inconspicuous [23] or not assessed [22] in these two studies. Hubacher et al. demonstrated that the training effect was sustained for nine months in both responders [22]. The sustainability of training effect was not assessed in the other two studies. Reported factors associated with outcomes of cognitive rehabilitation include various measures of disease burden, normalized brain volume, and general intelligence [22,23]. These are generally in line with the theory of brain reserve and cognitive reserve [25].

### 3.3. Registered Clinical Trials Primarily Focusing on Cognitive Issues in POMS

We searched on ClinicalTrials.gov, European Union Clinical Trials Register, and Open Science Framework database for pertinent trials or projects on 4 March 2020. Only three trials were considered most relevant. One trial aimed to explore the electrophysiological mechanisms underlying cognitive dysfunction in pediatric MS. The other was a randomized controlled trial assessing the efficacy of a home-based computerized program for retraining attention in pediatric patients with MS. These two trials were completed, and the results of the latter one has been published and included in the present review [24]. The third one is an ongoing randomized clinical trial aiming to assess the cognitive impact of a virtual reality videogame exercise program. More information about these trials is summarized in Table 4.

## 4. Discussions

Cognitive issues in pediatric MS have become a research priority in this field, and more studies surrounding cognitive evaluation for these patients were published over the past decade. Although routine cognitive screening is recommended for pediatric MS [26], and cognition has been incorporated into disease activity measure and treatment consideration [5,27,28], the best assessment tools for pediatric MS remain to be determined. Findings yielded by commonly used tools were sometimes discrepant across studies. For instance, SDMT has been recommended and widely used as a screening tool in adult and pediatric MS [26,29], whereas its sensitivity in pediatric MS was occasionally challenged [17,21,30,31], particularly in early stage of the disease. Brief international cognitive assessment for multiple sclerosis (BICAMS) and a Cogstate brief battery were shown to exhibit comparable sensitivity in detecting cognitive impairment in POMS [19]. These two batteries were subsequently employed in a study investigating the neuroanatomical correlates of cognitive impairment in POMS; however, the findings cast doubt on the discriminative power of BICAMS [31]. Trail making test (TMT)-B performance more readily differentiated between POMS and controls compared to TMT-A in earlier studies [13,30], whereas the reverse was found in later studies [21,32]. Bartlett et al. reported that verbal memory as assessed by Rey auditory verbal learning test (RAVLT) was impaired in POMS [31], while Storm Van’s Gravesande et al. found no significant verbal memory deficit in POMS using Verbaler Lern- und Merkfähigkeitstest (the German version of RAVLT) [21]. Discrepant results were also reported with regard to visuospatial memory tapped by brief visuospatial memory test-revised (BVMTR) versus Rey–Osterrieth figure tests [15,21]. Collectively, these reports underscore the need for more systematic evaluation on the selection and performance of neuropsychological tests in pediatric patients with MS.

Some of the discrepancies mentioned above could be due to differences in patient characteristics across studies. For example, the average EDSS score of subjects involved in evaluating multiple sclerosis inventory of cognition for adolescents (MUSICADO) was only 0.65, which might explain why verbal and visuospatial memory were preserved in POMS compared to controls in that study [21]. Indeed, Amato et al. reported that differences in verbal and visuospatial memory performance between POMS and healthy controls were inconspicuous at baseline [30], yet became significant at five-year follow-up [9]. Given that psychometric properties could be disease stage-dependent, and floor or ceiling effects could occur, longitudinal studies may be required to delineate the performance of various assessment tools along the disease course. This dimension has been less addressed so far, and existing studies were mostly cross-sectional in design.

Another issue concerns the validation of cognitive assessment tools in different populations and language versions, as was being performed for application of BICAMS in adults [33,34]. This is particularly important for pediatric populations, as many of the cognitive assessment tools have been shown to be age-sensitive during preteen to adolescent periods [14,15,17,21], and the relationship between age and cognitive development may be nonlinear [16]. In addition, most of existing studies were performed in North America and Europe, where some ethnic groups may be underrepresented [15]. Therefore, validation of the tools, including establishment of the norms, remain to be carried out in different populations.

Fatigue is relatively common in POMS, and it could be an important confounder for executive function or other aspects of cognitive performance in these patients [21,30,35,36,37,38]. Fatigue also poses practical limitation on the duration of assessment for these patients. It is intriguing to note that in the study evaluating computerized version of SDMT, POMS patients exhibited an “inverted U” pattern of performance over successive trials, in contrast to the progressive improvement observed in healthy controls [17]. This suggests a time-on-task effect, a psychological construct related to cognitive fatigue, which was often investigated using questionnaire [21,35]. A psychometric analysis of time-on-task effect in pediatric MS patients using cognitive tests involving repeated tasks (such as reaction time tasks) may deserve further research, as it could provide a complementary indicator of cognitive fatigue. The intra-individual variability, another neuropsychological metric of white matter pathology, could also be explored using these tasks [39]. Overall, more studies are needed to clarify whether and how fatigue and cognitive performance interact in pediatric MS.

On the other hand, our review shows that dedicated studies concerning cognitive rehabilitation for POMS remain scarce. A search for relevant clinical trials (Table 4) also showed a paucity of research specifically focusing on cognitive issues for pediatric MS, though this could be an underestimation because some trials may be retrospectively registered. Most current trials of cognitive rehabilitation for MS aim exclusively at adult populations [40,41]. We identified only three published studies evaluating effects of cognitive rehabilitation for POMS, two of which aimed to improve working memory and one targeted attention [22,23,24]. It is remarkable that all of these studies used home-based computerized programs. Although group- or institution-based rehabilitation may have merits in some circumstances, there appears a trend toward a more flexible and easy-to-access way of cognitive remediation, and these preliminary results seemed encouraging with regard to feasibility and patient satisfaction.

Concerning the impact of cognitive rehabilitation for POMS, these studies also showed promising results. More or less improvements in targeted cognitive domains were reported in all three studies, although discrepancy was noted between subjective and objective measures [23]. Given the heterogeneity and limited number of studies and their small sample size, no recommendation can be made for specific type of cognitive rehabilitation for POMS. It is noteworthy that the study on attention training showed far transfer effect [24], which is plausible because different facets of cognition could affect one another. These cross-modal effects deserve more exploration in future studies. Two studies of cognitive rehabilitation included neuroimaging evaluation [22,23], and one of them found correlation between working memory network activation and behavioral response [22], providing preliminary evidence that functional training is viable. Admittedly, more research is needed to resolve the controversial issue of functional training versus strategy training in cognitive rehabilitation for pediatric MS [42,43]. Sophisticated neuroimaging techniques may help to answer this question, as well as to clarify whether and how neuroplastic changes are facilitated by rehabilitation [44].

This scoping review has some limitations. First, we do not address social cognition, which appears more dissociable from other aspects of cognition and requires a separate approach [45,46]. Second, we do not examine studies of exercise training and its cognitive effects in pediatric patients with MS. There have been suggestions that physical activity may exert beneficial effects on cognition for both youth and MS patients, although more research in this direction is needed [47,48,49]. Third, given that our focus is on the cognitive assessment tools, we do not include studies aiming to characterize the cognitive profile of POMS. Nonetheless, we should acknowledge that in a broad sense many of those studies also contributed supportive evidence for the validity of various assessment tools.

## 5. Conclusions

Experiences with cognitive assessment tools for POMS are accumulating. Nonetheless, more research into their psychometric properties along the disease course may aid in the selection of appropriate tools during different disease stages. Computer-administered cognitive assessment and rehabilitation may be a trend worthy of further investigation. Systematic studies with larger sample size and rigorous methodology are much needed to inform evidence-based cognitive rehabilitation for POMS.

## Figures and Tables

**Table 1:** Overview of studies evaluating instruments for cognitive assessment for pediatric-onset multiple sclerosis (POMS).

Author (Year)	Test/Battery	Participants	Subtests and Targeted Cognitive Domains	Main Findings and/or Additional Notes
Portaccio et al. (2009) [13]	Brief Neuropsychological Battery for Children (BNBC)	61 POMS (age 8.8–17.9 years),58 matched HC	WISC vocabulary: language SDMT: processing speed, attention TMT: processing speed, attention, executive function SRT: verbal learning/memory	Sensitivity: 96%, specificity: 76% (cut-off: failure on at least one test) ~30 min
Smerbeck et al. (2011) [15]	Brief Visuospatial Memory Test–Revised (BVMTR)	51 POMS, 4 with ADEM, 92 HC (age 6–17)	visuospatial learning/memory	Regression-based pediatric norms Significantly poorer performance in pediatric patients with demyelinating disorders, with medium and large effect size for BVMTR (Cohen’s *d* −0.38~−0.71) and SDMT (Cohen’s *d* −1.30) respectively, between children with demyelinating disorders and HC.
	SDMT (oral version)	22 POMS, 3 with ADEM, 92 HC (age 6–17)	processing speed, attention	
Smerbeck et al. (2011) [20]	Brief Visuospatial Memory Test–revised (BVMTR)	43 POMS (age 9–18), 43 HC (age 9–18)	visuospatial learning/memory	significant difference between groups (Cohen’s *d* 0.9)
	SDMT (oral version)	20 POMS (age 9–18), 20 HC (age 8–18)	processing speed, attention	significant difference between groups (Cohen’s *d* 0.69)
Smerbeck et al. (2012) [16]	National MS Society Consensus Neuropsychological Battery for Pediatric Multiple Sclerosis (NBPMS)	51 POMS (age 9–18), 102 HC (age 5–18)	WASI: intelligence Grooved Pegboard Test: sensorimotor EOWPVT: language DKEFS Verbal Fluency: language Beery–Buktenika Test of Visual-Motor Integration: visuospatial processing CVLT-C: verbal learning/memory CPT-II: executive function, attention WISC-IV Digit Span: working memory WISC-IV Coding B: processing speed, attention Contingency Naming Test: executive function DKEFS TMT: executive function	Manual-based and regression-based (demographically adjusted) pediatric norms correlated strongly (*r >* 0.7) for all 30 variables. In 19 out of 30 variables, regression-based norms more readily detected neuropsychological impairment in POMS.
Charvet et al. (2014) [18]	Symbol Digit Modalities Test (SDMT, oral version)	70 POMS (70 underwent SDMT, 31 underwent neuropsychological testing), 40 other pediatric neurological diagnoses, 32 HC (note: significant difference in racial distribution between MS and HC)	processing speed, attention	SDMT showed 77% sensitivity and 81% specificity for neuropsychological impairment when the latter was done within one year, 100% sensitivity when the latter was done within two months. SDMT *z* score was significantly correlated with neuropsychological evaluation aggregate *z* score (*r* = 0.62, *p* < 0.001). Impaired SDMT performance in 37% of POMS and 9% of HC.
Bigi et al. (2017) [17]	Computer-Based Symbol Digit Modalities Test (c-SDMT)	27 POMS (22 female, 81.5%; age 8–18 years), 478 HC (237 female, 49.5%)	processing speed, attention	Regression analysis showed that increasing age (in the range 8–17) was significantly associated with better performance in HC. High test–retest reliability (ICC = 0.91) in HC. Total time to complete the task did not differ between POMS and HC, but POMS patients were less likely to show successively better performance over latter part of the task. Over 85% of participants (HC and POMS) indicated that they liked the test.
Charvet et al. (2018) [19]	Brief International Cognitive Assessment for Multiple Sclerosis battery (BICAMS)	69 POMS (7–21 years), 66 HC (8–21 years)	SDMT: processing speed, attention BVMTR: visuospatial learning/memory RAVLT: verbal learning/memory	Specificity: 91% Detection rate of cognitive impairment: 26% ~15 min
	Cogstate Brief Battery	67 POMS, 48 HC	Three speeded processing tasks: Detection: processing speed Identification: attention One-Back: working memory	Specificity: 92% Detection rate of cognitive impairment: 27% Detection and identification tasks (but not one-back) significantly discriminated between POMS and HC. ~15 min BICAMS and Cogstate agreed in the classification of impairment in 74% of the full sample (69% and 85% agreement for POMS and HC, respectively).
Kapanci et al. (2019) [8]	See subtests column (the study examined the interrelationships of tests tapping processing speed, working memory, and intelligence)	21 POMS, 21 matched HC	Reaction time task: processing speed Working memory task: working memory Cattell’s Culture Fair Test: intelligence	Intelligence measured by Cattell’s Culture Fair Test was significantly lower in POMS compared to HC. 33% of the variance in psychometric intelligence between POMS and HC was explained by differences in RT task performance. No difference in WM task performance between POMS and HC.
Brenton et al. (2019) [7]	See subtests column	20 POMS, 40 matched HC	SDMT: processing speed, attention PASAT (as a component of Multiple Sclerosis Functional Composite): processing speed, attention, working memory	POMS patients performed significantly lower on SDMT (*p* = 0.0002) and PASAT (*p* = 0.004). No significant correlation between SDMT *z* score and EDSS.
Falco et al. (2019) [14]	Rao’s Brief Repeatable Battery (BRB)	76 HC (age 14–17)	SRT and SRT-D: verbal learning/memory SPART and SPART-D: visuospatial learning/memory SDMT: processing speed, attention PASAT: processing speed, attention, working memory WLG: language (verbal fluency), executive function	Regression analysis showed that gender, age, and education were important variables in adolescent population. Younger age, male gender, and educational attainment were individually associated with better performance on SPART and SPART-D. Male gender was also associated with better performance on PASAT.
Storm Van’s Gravesande et al. (2019) [21]	Multiple Sclerosis Inventory of Cognition for Adolescents (MUSIC*ADO*)	106 POMS (age 12–18 years), 210 HC	Phonemic verbal fluency task (RWT “s-words”): executive function, language (verbal fluency) TMT-A: processing speed, attention Digit Span Forward: working memory	The phonemic verbal fluency task (RWT “s-words”), TMT-A, and Digit Span Forward tasks discriminated significantly between POMS and HC (*p* < 0.001, respectively). Specificity of MUSIC*ADO*: 88.6% Failure rate in POMS: RWT “s-words” 24.5%; TMT-A 17.9%; Digit Span Forward 15.1%.

Abbreviations: ADEM, acute disseminated encephalomyelitis; BICAMS, brief international cognitive assessment for multiple sclerosis battery; BVMTR, brief visuospatial memory test–revised; CPT-II, Conner’s continuous performance test—second edition; CVLT-C, California verbal learning test for children; DKEFS, Delis–Kaplan executive function system; EDSS, expanded disability status scale; EOWPVT, expressive one word picture vocabulary test; HC, healthy controls; ICC, intraclass correlation coefficient; PASAT, paced auditory serial addition test; POMS, pediatric-onset multiple sclerosis; RAVLT, Rey auditory verbal learning test; RT, reaction time; RWT, Regensburger Wortflüssigkeitstest; SDMT, symbol digit modalities test; SPART, spatial recall test; SPART-D, SPART delayed recall; SRT, selective reminding test; SRT-D, SRT delayed recall; TMT, trail-making test; WASI, Wechsler abbreviated scales of intelligence; WISC-IV, Wechsler intelligence scale for children—fourth edition; WLG, word list generation; WM, working memory.

**Table 2:** Overview of studies evaluating cognitive rehabilitation for POMS: Study design.

Author (Year)	Intervention (Duration and Frequency)	Supervision or Coaching	Targeted Cognitive Domain	Study Participants	Comparator Group	Outcome Measures
Hubacher et al. (2015) [22]	computerized training (BrainStim) for 4 weeks (45 min/session, 4 times/week)	supervised by a psychologist once per week	working memory (visuospatial and verbal)	5 juvenile MS patients (age 12–17 years; 3 females)	Absent	Cognitive measures: working memory (visuospatial and verbal) and attention (alertness)
Simone et al. (2018) [24]	computerized training for 3 months (1 h/session, twice/week)	a psychologist called patients every week and met patients and their caregiver/parent every month	attention	16 POMS patients (age 15.75 ± 1.74 years; 9 females)	Present (nonspecific training)	Neuropsychological performance (using elaborate test battery)
Till et al. (2019) [23]	web-based training (Cogmed™) for 5 weeks (<1 h/session, 5 d/week)	weekly telephone support by a trained Cogmed™ Coach	working memory (visuospatial and verbal)	9 POMS patients (age 19.3 ± 4.1 years; 6 females)	Absent	Feasibility measures: adherence and tolerance; Cognitive measures: working memory, processing speed, visuospatial judgment

**Table 3:** Original studies evaluating cognitive rehabilitation for POMS: Summary of results.

Author (Year)	Intervention	Effects on Targeted or Related Cognitive Domains	Far Transfer Effect	Sustainability of Effect	Factors Associated with Training Response, and Additional Notes
Hubacher et al. (2015) [22]	computerized training (BrainStim) for 4 weeks (45 min/session, 4 times/week)	Two (of 5) were responders; both responders showed better WM (visuospatial and verbal), processing speed, and alertness.	Not assessed	Sustained behavioral response at 9 months in both responders	Disease activity and general intelligence may be factors associated with training response.
Simone et al. (2018) [24]	computerized training for 3 months (1 h/session, twice/week)	Improved attention, processing speed, and WM.	Improved executive function and visuospatial memory	NA	Not reported
Till et al. (2019) [23]	web-based training (Cogmed™) for 5 weeks (<1 h/session, 5 d/week)	Subjective: 8 (out of 9) reported improvement in WM; Objective: medium to large effect size on neuropsychological measures of WM.	Limited	NA	Indicators of feasibility: 6/9 adherence; 8/9 tolerance. The participant who showed the least improvement had the youngest age at disease onset, longest disease duration, highest number of relapses, and lowest normalized brain volume. The participant who did not tolerate the training had the lowest IQ.

Abbreviations: NA, not available; WM: working memory.

**Table 4:** Registered clinical trials primarily focusing on cognitive issues in POMS.

ClinicalTrials.Gov Identifier	NCT03066752	NCT03190902	NCT03933020
Aim	To study the neural mechanisms underlying cognitive dysfunction	To assess the efficacy of a computerized program for retraining attention	To assess the cognitive impact of a home-based virtual reality videogame exercise program
Study period	March~November 2017	September 2015~April 2016	May 2019~June 2020
Study type	Observational	Interventional	Interventional
Trial design	Prospective case-control	Double blind, randomized clinical trial	Single blind, randomized clinical trial
Ages eligible for study	6~18 years	up to 17 years	15~25 years
Recruitment status	Completed	Completed	Recruiting
Enrollment	10 cases, 10 controls (actual)	8 cases, 8 controls (actual)	12 cases, 12 controls (estimated)
Principal investigator	E. Ann Yeh	Pietro Iaffaldano	Stephanie Garcia-Tarodo

## Supplementary Materials

The following are available online at https://www.mdpi.com/2227-9067/7/10/183/s1, Supplementary File S1: PRISMA-ScR Checklist, Supplementary File S2: Scoping Review Protocol.
