# Supplementary material for: Cognitive Assessment and Rehabilitation for Pediatric-Onset Multiple Sclerosis: A Scoping Review
Source: Children (Basel). 2020 Oct 15;7(10):183. doi: 10.3390/children7100183 (PMC7602453; doi:10.3390/children7100183)
Supplement: Supplementary file 1 [file children-07-00183-s001.zip › Supplementary file S2. Scoping Review Protocol.docx]

**Scoping Review Protocol: Cognitive assessment and rehabilitation for pediatric-onset multiple sclerosis**

**Background**

Cognitive impairment is increasingly recognized as an important clinical issue in pediatric-onset multiple sclerosis (MS). Cross-sectional studies showed that around one-third of pediatric patients with MS were affected in various aspects of cognition, including attention, working memory, language, executive function, and social cognition.^1-3^ However, research regarding cognitive assessment tool and cognitive rehabilitation for these patients remain limited in quantity and scale. Variations in clinical practice concerning cognitive evaluation and remediation are noted in these patients in Taiwan, presumably due to inadequacy of evidence-based guidelines. Therefore, we propose this scoping review to address the questions specified below.

**Review questions**

This scoping review seeks to clarify, through the available literature, the following questions:

(1) What are the psychometric properties and feasibility of various cognitive assessment tools, alone or in combination, in the context of pediatric-onset MS?

(2) What constitutes current best practice with regard to cognitive interventions for patients with pediatric-onset MS?

**Inclusion criteria**

Types of participants:

This review will consider all studies that involve human participants with pediatric-onset MS.

Types of studies:

(1) Peer-reviewed original studies published in English;

(2) Studies that specifically address either the psychometric properties of cognitive assessment tools or the feasibility and/or efficacy/effectiveness of cognitive rehabilitation for pediatric-onset MS.

**Search strategy**

We will conduct systematic literature searches in the following databases: PubMed, MEDLINE, CINAHL Plus, and Web of Science. Date of publication is not restricted (from inception to February 2020). The terms used in the searches are: “multiple sclerosis” AND (“pediatric” or “paediatric”) AND (“cognitive” or “cognition”). The titles and abstracts of retrieved articles will then be screened by two reviewers independently for relevance to our review questions.

**Data collection**

The included articles will be read by two reviewers independently, and relevant data will be charted and tabulated. For studies evaluating cognitive assessment tools, we aim to extract the year of publication, the tests (and subtests, if applicable) of interest and their targeted cognitive domains, the characteristics of study participants (such as sample size of disease and control groups, demographic and disease-related features), and main findings (particularly in relation to psychometric performance). For studies evaluating cognitive rehabilitation, we aim to extract the mode of intervention (including its frequency and duration, requirement of supervision, and targeted cognitive domains), study design, the characteristics of study participants, effects of intervention (including effects on targeted and non-targeted domains, and sustainability of effect), and factors associated with outcomes.

**Data synthesis**

For quantitative data, odds ratio (for categorical outcome data) or standardized mean differences (for continuous data) and their 95% confidence intervals will be calculated from the data derived from each included randomized controlled trial. When statistical pooling is inappropriate or not possible, the findings will be presented in narrative form.

**References**

1. Parrish JB and Fields E. Cognitive Functioning in Patients with Pediatric-Onset Multiple Sclerosis, an Updated Review and Future Focus. *Children (Basel)* 2019; 6 2019/02/06. DOI: 10.3390/children6020021.

2. Charvet LE, Cleary RE, Vazquez K, et al. Social cognition in pediatric-onset multiple sclerosis (MS). *Mult Scler* 2014; 20: 1478-1484. 2014/03/22. DOI: 10.1177/1352458514526942.

3. Amato MP, Krupp LB, Charvet LE, et al. Pediatric multiple sclerosis: Cognition and mood. *Neurology* 2016; 87: S82-87. 2016/08/31. DOI: 10.1212/wnl.0000000000002883.
